# Symptoms of post-traumatic stress disorder (PTSD) in next of kin during suspension of ICU visits during the COVID-19 pandemic: a prospective observational study

**DOI:** 10.1186/s12888-021-03468-9

**Published:** 2021-09-29

**Authors:** Bjoern Zante, Katja Erne, Julia Grossenbacher, Sabine A. Camenisch, Joerg C. Schefold, Marie-Madlen Jeitziner

**Affiliations:** 1 Department of Intensive Care Medicine, Inselspital, Bern University Hospital, University of Bern, Freiburgstrasse 10, 3010 Bern, Switzerland; 2 Department of Anesthesiology and Pain Medicine, Inselspital, Bern University Hospital, University of Bern, Bern, Switzerland; 3 Department of Public Health, Nursing Science, Faculty of Medicine, University of Basel, Basel, Switzerland

**Keywords:** Critical care, Post intensive care syndrome–family, Mental distress, Stress disorder, Psychological disease, Relatives

## Abstract

**Background:**

During the COVID-19 pandemic, suspension of visits by next of kin to patients in intensive care units (ICU), to prevent spread of the SARS-CoV-2 virus, has been a common practice. This could impede established family-centered care and may affect the mental health of the next of kin. The aim of this study was to explore symptoms of post-traumatic stress syndrome (PTSD) in the next of kin of ICU patients.

**Methods:**

In this prospective observational single-center study, next of kin of ICU patients were interviewed by telephone, using the Impact of Event Scale-Revised (IES-R), to assess symptoms of acute stress disorder during the ICU stay and PTSD symptoms at 3 months after the ICU stay. The primary outcome was the prevalence of severe PTSD symptoms (IES-R score ≥ 33) at 3 months. The secondary outcomes comprised the IES-R scores during the ICU stay, at 3 months, and the prevalence of severe symptoms of acute stress disorder during ICU stay. An inductive content analysis was performed of the next of kin’s comments regarding satisfaction with patient care and the information they were given.

**Results:**

Of the 411 ICU patients admitted during the study period, 62 patients were included together with their next of kin. An IES-R score > 33 was observed in 90.3% (56/62) of next of kin during the ICU stay and in 69.4% (43/62) 3 months later. The median IES-R score was 49 (IQR 40–61) during the ICU stay and 41 (IQR 30–55) at 3 months. The inductive content analysis showed that communication/information (55%), support (40%), distressing emotions (32%), and suspension of ICU visits (24%) were mentioned as relevant aspects by the next of kin.

**Conclusions:**

During the suspension of ICU visits in the COVID-19 pandemic, high prevalence and severity of both symptoms of acute stress disorder during the ICU stay and PTSD symptoms 3 months later were observed in the next of kin of ICU patients.

**Supplementary Information:**

The online version contains supplementary material available at 10.1186/s12888-021-03468-9.

## Background

Post-traumatic stress disorder (PTSD) may develop after exposure to a single or multiple traumatic event(s) and/or prolonged traumatic event [1]. The published figures for life-time prevalence vary between 1.9 and 7.8% [2, 3].

The prevalence of clinically relevant PTSD symptoms among family members of patients admitted to the intensive care unit (ICU) has been estimated at 33 to 56% [4–7]. Sparse provision of family-centered care (e.g., timely provision of adequate information, involvement in decision-making, social and emotional support, next of kin’s abilities to provide care) in the ICU may play a crucial role in the development of PTSD symptoms [7]. Interventions to support next of kin of ICU patients during the ICU stay and there after often prove insufficient [8].

On March 11, 2020, the World Health Organization (WHO) characterized the worldwide outbreak of COVID-19 as a pandemic. Various measures, including social distancing, were introduced to reduce the spread of the SARS-CoV-2 virus. As recently shown, this pandemic has affected adult mental health; reports of the prevalence of clinically relevant PTSD symptoms range from 15.8 to 37.7% [9–11].

In many ICUs worldwide, visits were suspended to comply with social distancing requirements and prevent the spread of contamination among staff, patients, and next of kin. This practice may impede the provision of family-centered care in ICUs [12, 13]. Therefore, we hypothesize that patients’ next of kin may have been exposed to a higher risk of PTSD symptoms during the COVID-19 pandemic [14]. This could have detrimental effects on health, social life, and working life [15].

We therefore set out to investigate the prevalence and experience of PTSD symptoms in the next of kin of ICU patients during suspension of ICU visits in the COVID-19 pandemic. Hence, we interviewed next of kin of ICU patients by telephone, using the Impact of Event Scale-Revised (IES-R), to assess symptoms of acute stress disorder during the ICU stay and PTSD symptoms 3 months thereafter, with qualitative content analysis of their comments and experiences.

## Methods

### Study design

A prospective single-center study of the next of kin of ICU patients was performed at a tertiary academic center. The study was conducted in the interdisciplinary (medical/surgical) ICUs of the Department of Intensive Care Medicine at the Inselspital in Bern, Switzerland between March 16 and May 11, 2020. During this observation period, all hospital visits by next of kin were suspended to prevent spreading of the SARS-CoV-2 virus among staff, patients, and their family members. Exceptions were made for end-of-life visits.

### Participants

The participants in this study were the next of kin of patients hospitalized in the ICU during the observation period. The exclusion criteria were patient/next of kin aged < 18 years; inability of next of kin to participate in a telephone interview due to insufficient knowledge of German, French, or Italian; refusal by next of kin to participate in the study; and failure to make contact with next of kin before patient’s discharge from the ICU.

### Ethics

The Ethics Committee on Human Research, Bern, waived the requirement for ethics approval and the need to obtain consent for the collection, analysis, and publication of the data for this study (KEK Req-2020-00739). However, oral informed consent was obtained from the participating next of kin. This study adhered to the tenets of the Declaration of Helsinki.

### Outcome measures

In brief, for each individual patient who fulfilled the inclusion and exclusion criteria for this study, the next of kin was identified at admission to the ICU. The next of kin who met the inclusion criteria were interviewed twice by telephone, to assess potential symptoms of acute stress disorder at 1–5 days after patient ICU admission and PTSD symptoms at 3 months after discharge from or death in the ICU (as defined in the *Statistical Manual of Mental Disorders*; DSM-5) [16].

The validated IES-R was used to estimate symptoms of acute stress disorder at day 1–5 [17–19] and PTSD symptoms after 3 months [20–26]. The IES-R is a 22-item questionnaire with three defined subscales (avoidance, intrusion, and hyperarousal). Items were rated on a five-point Likert scale from 0 (“not at all”) to 4 (“extremely”), yielding a total score ranging between 0 (best) and 88 (worst). We chose an IES-R score of 33 as cut-off for severe PTSD symptoms, in line with previous studies [22, 24].

Family satisfaction in the ICU was assessed using an adapted version of the Family Satisfaction in the Intensive Care Unit 24-Item-Revised (FS-ICU 24R) questionnaire [27]. The questionnaire was adapted to the suspension of ICU visits by exclusion of questions that could not be answered without the physical presence of the next of kin in the ICU (see additional file 1). Items were rated on a five-point Likert scale. The scale for items 1 to 8 ranged from 1 (“very dissatisfied”) to 5 (“completely satisfied”), while the scale for item 9 ranged from 1 (“I felt very excluded”) to 5 (“I felt very included”). The final score of the adapted FS-ICU 24R ranged from 9 (worst) to 45 (best). As proposed in the original scoring manual, responses on the adapted version were excluded whenever > 30% of items were missing (*n* = 9/62,14%) [28].

The patient data recorded included demographics, emergency admission, Acute Physiology and Chronic Health Evaluation II (APACHE II) score, Simplified Acute Physiology Score II (SAPS II), COVID-19 positivity, mechanical ventilation, length of stay, and death in the ICU. The demographics of the next of kin were also recorded. All data recorded are parameters that may contribute to symptoms of acute stress disorder and/or PTSD symptoms in the next of kin [7].

The primary outcome was the prevalence of severe symptoms of PTSD (defined as IES-R score > 33) among the next of kin at 3 months after patient discharge from the ICU (or death) [22, 24]. The secondary outcomes included the next of kin IES-R scores (range 0–88) at 3 months, the proportion with an prevalence of severe PTSD symptoms (IES-R score > 33,) and total IES-R score (0–88) during ICU stay.

### Qualitative data

An inductive content analysis was performed on the next of kin’s comments and experience regarding general satisfaction with care and satisfaction with decision-making [29]. This content analysis was embedded in the adapted FS-ICU 24R questionnaire, and data were used for hypothesis generation with regard to factors potentially contributing to symptoms of acute stress disorder, mental stress and PTSD symptoms. Two researchers (MMJ, KE) with experience in inductive content analysis evaluated the comments.

### Statistical analysis

Categorical variables were computed as proportions; continuous variables as median and interquartile range (IQR) or mean and standard deviation (SD), as appropriate. Normal distribution was checked using the Shapiro-Wilk test. Comparisons of categorical variables between groups (defined by IES-R cut-off values) were performed with Fisher’s exact test, and continuous variables were compared using the Mann–Whitney U-test or the t-test, as appropriate. The McNemar test and the Wilcoxon rank-sum test were used to compare IES-R categorical and continuous data between the ICU stay and the 3-month follow-up. A two-tailed *p*-value < 0.05 was considered to show a significant difference. Statistical analyses were performed using SPSS version 25 (IBM Corp, 2017) and R (R Core Team, version 4.0.3).

If two or fewer answers were missing in the IES-R scale (*n* = 4 in the ICU survey, *n* = 3 in the 3-month survey) the missing responses were substituted using the *person mean substitution* method [30]. Data sets with three or more answers missing were excluded from analysis (*n* = 10).

## Results

### Study population

**Fig. 1:**
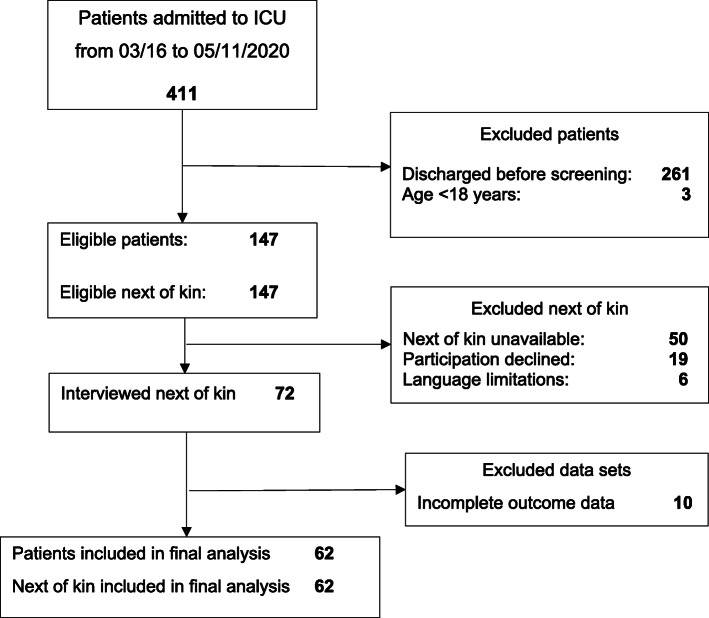
Screening and inclusion of next of kin

Of the 411 patients admitted to the ICU during the observation period, 62 patients and their next of kin were included in the study (Fig. 1). Among the 261 patients discharged prior to screening, 212 had an ICU stay of less than 24 h. The demographic characteristics of next of kin and patients are given in Tables 1 and 2. No differences in the demographic data of next of kin and patients were noted between the two IES-R groups (IES-R < 33 vs. IES-R > 33) during the ICU stay (Table 3). In addition, no differences in the demographic data of next of kin and patients were observed between the IES-R groups at 3 months’ follow-up, with the exception of admission to the ICU after surgery (*p* = 0.03; Table 4).

**Table 1:** Characteristics of next of kin

	*N*	Value
Age, years	62	58 (50–70)
Female gender, no. (%)	62	50 (81)
IES-R score in ICU	62	49 (40–61)
IES-R score at follow-up	62	41 (30–55)
Family satisfaction (FS-ICU 24R)	53	33 (28–40)
Family members, no. (%)	62	60 (96.8)

Age, IES-R score, and family satisfaction are expressed as median and interquartile range

**Table 2:** Characteristics of ICU patients

	*N*	Value
Age, years	62	69 (53–75)
Female gender, no. (%)	62	14 (22.6)
Emergency admission, no. (%)	62	52 (83.9)
Surgical base diagnosis, no. (%)	62	37 (59.7)
APACHE II	61	25.8 + 7.9
SAPS II	62	49.3 + 17.4
COVID-19 positive, no. (%)	62	8 (12.9)
Admission after surgery, no. (%)	62	27 (43.6)
Mechanical ventilation, no. (%)	62	44 (71.0)
Duration of mechanical ventilation, hours	44	32.8 (10.9–111.9)
ICU length of stay, days	62	2.87 (1.1–8.4)
Died in ICU, no. (%)	62	4 (6.5)

Age, duration of mechanical ventilation, and length of stay are expressed as median and interquartile range; the Acute Physiology and Chronic Health Evaluation II (APACHE II) score and the Simplified Acute Physiology Score II (SAPS II) are expressed as mean and standard deviation

**Table 3:** Comparison of characteristics related to IES-R score in ICU

Characteristic	IES-R < 33	IES-R **>** 33	***P***-value
**Next of kin**	*n* = 6	*n* = 56	
Age	61 (50–75)	58 (50–70)	0.65
Female gender, no. (%)	5 (83.3)	45 (80.4)	1.0
IES-R score	32 (27–32)	54 (41–62)	< 0.001
Family satisfaction (FS-ICU 24R)	30 (27–39)	34 (28–40)	0.63
Family members, no. (%)	5 (83.3)	55 (98.2)	0.18
**Patients**			
Age	74.5 (60.5–75.3)	68 (50–75)	0.59
Female gender, no. (%)	2 (33.3)	12 (21.4)	0.61
Emergency admission, no. (%)	4 (66.7)	48 (85.7)	0.25
Surgical base diagnosis, no. (%)	4 (66.7)	33 (58.9)	1.0
APACHE II	21.67 + 9.5	26.2 + 7.7	0.19
SAPS II	44.2 + 17.3	49.8 + 17.5	0.46
COVID-19 positive, no. (%)	0 (0)	8 (14.3)	1.0
Admission after surgery, no. (%)	4 (66.7)	23 (41.1)	0.23
Mechanical ventilation, no. (%)	4 (66.7)	39 (69.6)	1.0
Duration of mechanical ventilation, hours	16.6 (3–39.7)	34.7 (11.8–140.2)	0.8
ICU length of stay, days	1.6 (0.8–3.9)	3.27 (1.1–10.2)	0.14
Died in ICU, no. (%)	0 (0)	4 (7.1)	1.0

Next of kin’s age, IES-R score, family satisfaction score, patients’ age, duration of mechanical ventilation, and length of stay are expressed as median and interquartile range; the Acute Physiology and Chronic Health Evaluation II (APACHE II) score and the Simplified Acute Physiology Score II (SAPS II) are expressed as mean and standard deviation

**Table 4:** Comparison of characteristics related to IES-R score at 3 months

Characteristic	IES-R < 33	IES-R **>** 33	***P***-value
**Next of kin**	**19**	**43**	
Age	62 (50–73)	57 (51–69)	0.6
Female gender, no. (%)	16 (84.2)	34 (79.1)	0.74
IES-R score	27 (26–30)	48 (38–60)	< 0.0001
Family satisfaction (FS-ICU 24R)	33 (30–39)	34 (25–40)	0.9
Family members, no. (%)	18 (94.7)	42 (97.7)	0.52
**Patients**			
Age	67 (59–74)	69 (48–76)	0.77
Female gender, no. (%)	6 (31.6)	8 (18.6)	0.33
Emergency admission, no. (%)	14 (73.7)	38 (88.4)	0.26
Surgical base diagnosis, no. (%)	13 (68.4)	24 (55.8)	0.41
APACHE II	24 + 8.9	26.6 + 7.4	0.25
SAPS II	48.4 + 16.6	49.7 + 17.9	0.79
COVID-19 positive, no. (%)	1 (5.3)	7 (16.3)	0.42
Admission after surgery, no. (%)	12 (63.2)	15 (34.9)	0.03
Mechanical ventilation, no. (%)	12 (63.2)	31 (72.1)	0.56
Duration of mechanical ventilation, hours	26.6 (8.5–97.7)	36.1 (10.9–155.3)	0.46
ICU length of stay, days	2.8 (0.9–8.2)	3.2 (1.3–8.7)	0.55
Died in the ICU, no. (%)	0 (0)	4 (9.3)	0.3

Next of kin’s age, IES-R score, family satisfaction score, patients’ age, duration of mechanical ventilation, and length of stay are expressed as median and interquartile range; the Acute Physiology and Chronic Health Evaluation II (APACHE II) score and the Simplified Acute Physiology Score II (SAPS II) are expressed as mean and standard deviation

### Prevalence of symptoms of acute stress disorder and PTSD symptoms in next of kin of ICU patients during suspension of ICU visits

An IES-R score > 33 was observed in 90.3% (*n* = 56/62) of next of kin during the patients’ ICU stays and in 69.4% (*n* = 43/62) of next of kin 3 months after ICU discharge. The median IES-R score was 49 (IQR 40–61) in the ICU and 41 (IQR 30–55) at the 3-month follow-up (Table 1). A difference was observed between the frequency of severe symptoms of acute stress disorder (cut-off IES-R > 33) during the ICU stay (*n* = 56) and the frequency of severe PTSD symptoms at follow-up 3 months after discharge (*n* = 43; *p* = 0.001). The IES-R scores declined between the ICU stay and the 3-month follow-up (from 49 (IQR 40–61) to 41 (IQR 30–55); *p* < 0.0001).

### Prevalence of symptoms of acute stress disorder and PTSD symptoms in next of kin subgroups of next of kin

Overall, 43.6% (27/62) of the patients were admitted to the ICU following surgical interventions (Table 2). No differences were observed in patients’ admissions after emergency surgery comparing next of kin with IES-R score < 33 and IES-R score > 33 (IES-R score < 33 vs. IES-R score > 33; *n* = 2/4, 50%; *n* = 15/23, 65.2%, respectively, *p* = 0.61,) neither in the ICU nor at 3 months (*n* = 7/12, 58.3%; *n* = 10/15, 66.7%, respectively, *p* = 0.71).

Information on the educational level was available in 75.8% of the next of kin (*n* = 47/62). Higher educational level (higher training/ university degree) was found in 29.8% (*n* = 14/47). No difference was observed regarding IES-R score (< 33 or > 33) between next of kin with and those without higher educational training, neither in the ICU (*n* = 3/5, 60% vs. *n* = 11/42, 26.2%; *p* = 0.15) or at 3 months follow-up (*n* = 6/16, 37.5% vs. *n* = 8/31, 25.8%; *p* = 0.5).

### Overall family satisfaction

**Fig. 2:**
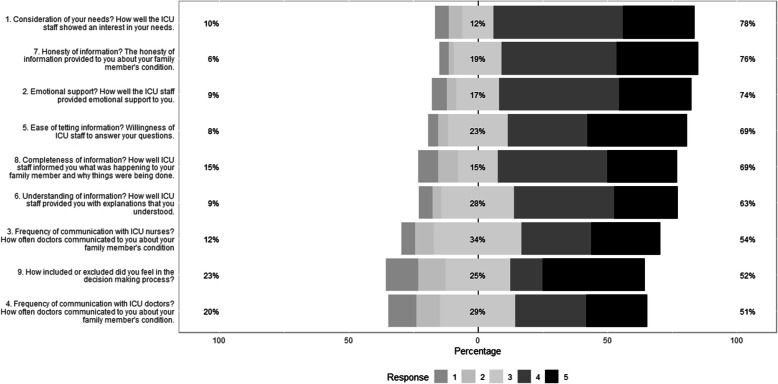
Proportions of answers on the adapted FS-ICU 24R ordered by degree of agreement. Responses for items 1 to 8 were rated on a five-point Likert scale ranging from 1 (“very dissatisfied”) to 5 (“completely satisfied”), while the scale for item 9 ranged from 1 (“I felt very excluded”) to 5 (“I felt very included”)

Family satisfaction was assessed using an adapted version of the FS-ICU 24R, and a median score of 33 (IQR 28–40; Table 1) was observed. No differences were observed between the two IES-R groups (IES-R score < 33 vs. > 33) during ICU stay (*p* = 0.63; Table 3) or at 3 months (*p* = 0.9; Table 4). The distribution of answers on the adapted FS-ICU 24R is shown in Fig. 2.

### Qualitative content analysis

Overall, 156 comments from the 62 next of kin were evaluated in an inductive content analysis. Four main categories of experiences were elaborated from their comments: (1) communication and information (*n* = 34/62, 55%); (2) support received (*n* = 25/62, 40%); (3) distressing emotions (*n* = 20/62, 32%); (4) suspension of ICU visits (*n* = 15/62, 24%).

Aspects of communication and information appeared most important to the next of kin. Positive feedback was associated with no limitations being placed on time, active telephone calls from ICU staff, frankness about the specific situation, avoidance of technical terminology, repetition of information, and establishment of the current state of knowledge at the beginning of conversations. Factors cited as reasons for negative experiences included language barriers, decreased comprehensibility, and constraints on time to talk with the patient.

With regard to support, the next of kin appreciated the availability of video telephone calls and/or provision of ICU diaries (which forms part of the institutional standard of family-centered care) including photographs and explanations of the patient’s status and specific ICU setting. Further, questions regarding the well-being of the next of kin and provision of information about daily ICU care and routines (e.g., mobilization, weaning trials, interventions) elicited positive comments.

In the third category, the next of kin highlighted distressing emotions related to the situation and reported feelings of anxiety, worry, and loneliness. As for the fourth category, suspension of ICU visits, some next of kin described the situation as “terrible” because they “lost contact with loved ones”. However, we noted that the next of kin attempted to cope with specific situations and the respective consequences, including suspension of ICU visits.

## Discussion

In a single-center prospective study, we investigated the prevalence of symptoms of acute stress disorder during the ICU stay and PTSD symptoms 3 months thereafter among the next of kin of ICU patients during suspension of ICU visits due to the COVID-19 pandemic. We observed that the majority of the next of kin experienced severe symptoms of acute stress disorder during their family member’s ICU stay and severe PTSD symptoms afterwards. Previous studies had reported that the next of kin of ICU patients may experience symptoms of PTSD [7]; however, we observed potentially higher prevalence and greater severity of PTSD symptoms during the COVID-19 pandemic [4–6].

A number of factors may be responsible for potentially higher prevalence and severity of the symptoms of acute stress disorder/PTSD symptoms among the next of kin of ICU patients in this specific context. First, it appears that the COVID-19 pandemic itself induces PTSD [9–11]. Among other factors, uncertainty, confusion, and anxiety regarding infection and the social consequences may increase the likelihood of symptoms of acute stress disorder and/or PTSD symptoms [9, 11]. This “baseline” psychological distress may be increased in the next of kin of ICU patients [7], with potential augmentation of existing symptoms of acute stress disorder and/or PTSD symptoms [31, 32].

Other investigations have proposed that the risk of developing PTSD symptoms may be associated with particular demographic characteristics (e.g., female gender, age of next of kin and/or patient, educational level) [33–35]. In our study, however, no association between specific demographic data and the development of severe PTSD symptoms was observed. Further, levels of communication and information provision seemed to be risk factors for PTSD development [6, 36, 37]. Interestingly, despite good ratings for the next of kin’s satisfaction with regard to information provided, the prevalence and severity of PTSD symptoms among them remained high. This may imply that provision of information during the suspension of ICU visits meets the requirements of family-centered care [12].

Indeed, information can still be provided during suspension of ICU visits [13, 38, 39]. Other components of family-centered care can also be delivered in alternative ways at such a time [13, 14, 40]. However, the effects on symptoms of acute stress disorder/PTSD symptoms among next of kin remain unclear [8]. Indeed, inadequate provision of emotional support [41, 42] and insufficient communication regarding the patient’s prognosis [43, 44] and shared decision-making [45] may be related to PTSD symptoms. However, the importance of the physical presence of the next of kin for these specific family-centered care components remains unclear.

The qualitative data (inductive content analysis) may appear to imply that suspension of visits is challenging for next of kin. It is therefore tempting to speculate that suspension of ICU visits amplifies uncertainty regarding comfort and the specific patient situation in the ICU [6, 14, 46]. Investigations into liberal visiting policies in the ICU have revealed a positive impact on family satisfaction; however, the impacts on anxiety, depression, and PTSD symptoms remained unclear [47]. Hence, the content and context of the ICU visits may be crucial for coping with PTSD symptoms (avoidance of frustration, improvement of vigilance, reassurance, proximity, provision of information) [48, 49].

The next of kin’s symptoms of acute stress disorder may be explained by the exceptional situation due to the patients’ ICU stay. Importantly, this acute stress disorder should subside within days [50]. This may partly explain the decline of the IES-R score. However, despite a decline in the prevalence and severity of the PTSD stress symptoms relative to the time of the ICU stay, the respective IES-R scores remained high 3 months later. Certainly, the mental stress caused by the COVID-19 pandemic seems to continue to this time. Likewise, the stress arising from ICU hospitalization may still be noticeable [4, 6]. These two factors appear to contribute to the severe PTSD symptoms in the next of kin of ICU patients [31, 32]. Notably, although the next of kin who exhibit transient symptoms of an acute stress disorder within days may not necessarily receive the diagnosis of PTSD after 3 month or later [51].

This study has several important limitations. The monocentric study design with a limited sample size and the imbalance between the groups compared may lead to a reduced external validity, especially in the context of family-centered care concepts. Nevertheless, the strengths of our study include the prospective design and the qualitative content analysis. Importantly, the IES-R is not suitable for the diagnosis of PTSD as defined in the *Statistical Manual of Mental Disorders* (DSM-5). To diagnosis PTSD, a clinician-administered structured clinical assessment is needed. Hence, in this study only symptoms of acute stress disorder and PTSDrelated symptoms could be estimated, with all the inherent limitations of a questionnaire**.** The IES-R was originally used to estimate PTSD symptoms [22]. Therefore, we used the IES-R, during the traumatic event (ICU hospitalization of a family member) for the sake of better comparability and progression of the symptoms among next of kin during and after the event [17–19]. This should be taken into consideration when interpreting the results. Moreover, no information was available on quarantine and/or isolation, which could theoretically impact on PTSD symptoms. However, the data on this topic remain controversial [9–11].

Furthermore, no data were available on the potential role of missing social support, which could affect PTSD symptoms [10, 52–54]. Also, despite good provision of information on the patients’ condition in the ICU, the impact of information about the COVID-19 pandemic, which itself could contribute to PTSD symptoms, should not be neglected [10]. Information on pre-existing psychological disorders and/or PTSD symptoms was not available for the next of kin; hence, the potential impact of any such factors remains unclear. In addition, our data show an imbalance in the gender distribution of the next of kin (more women), and previous studies indicate that women may be more susceptible to PTSD symptoms, both in general and in the context of the COVID-19 pandemic [9–11, 55, 56].

## Conclusions

During the suspension of ICU visits in the COVID-19 pandemic, high prevalence and severity of both symptoms of acute stress disorder during the ICU stay and PTSD symptoms 3 months after discharge or death were observed in the next of kin of ICU patients. Additional investigations are required to investigate factors involved in PTSD and symptom development in the next of kin of ICU patients during the COVID-19 pandemic. Furthermore, interventional strategies should be developed to provide specific support to next of kin both during and after the patient’s ICU stay.

## Supplementary Information

**Additional file 1: Table S1.** Items of the adapted FS-ICU-24R. **Table S2.** Descriptive analysis of the adapted FS-ICU-24R. **Table S3.** Item loadings of the adapted FS-ICU-24R. **Fig. S1.** Scree plot.

## Data Availability

All data generated and/or analyzed during this study are included in this published article (and in its supplementary information file).

## Abbreviations

PTSD: Post-traumatic stress disorder
ICU: Intensive care unit
IES-R: Impact of event scale-revised
IQR: Interquartile range
WHO: World health organization
KEK: *Kantonale ethikkommission* (regional ethics committee)
FS-ICU 24R: Family satisfaction in the intensive care unit 24-item-revised
SD: Standard deviation
APACHE II: Acute physiology and chronic health evaluation II
SAPS II: Simplified acute physiology score
