# Supplementary material for: Symptoms of post-traumatic stress disorder (PTSD) in next of kin during suspension of ICU visits during the COVID-19 pandemic: a prospective observational study
Source: BMC Psychiatry. 2021 Sep 29;21:477. doi: 10.1186/s12888-021-03468-9 (PMC8480126; doi:10.1186/s12888-021-03468-9)
Supplement: Supplementary file 1 — Additional file 1: Table S1. Items of the adapted FS-ICU-24R. Table S2. Descriptive analysis of the adapted FS-ICU-24R. Table S3. Item loadings of the adapted FS-ICU-24R. Fig. S1. Scree plot. [file 12888_2021_3468_MOESM1_ESM.docx]

**Symptoms of post-traumatic stress disorder (PTSD) in next of kin during suspension of ICU visits during the COVID-19 pandemic: a prospective observational study**

Bjoern Zante MD MME^1^, Katja Erne MScPsychol^1^, Julia Grossenbacher BscN^1^, Sabine A. Camenisch MD^2^, Joerg C. Schefold MD^1^, Marie-Madlen Jeitziner PhD RN^1^,

1. Department of Intensive Care Medicine, Inselspital, Bern University Hospital, University of Bern, Bern, Switzerland
2. Department of Anesthesiology and Pain Medicine, Inselspital, Bern University Hospital, University of Bern, Switzerland.

**Supplemental content**

Principal component analysis, confirmatory factor analysis and internal consistency calculation for the adapted FS-ICU-24R

Family satisfaction in the ICU was measured with an adapted version of the Family Satisfaction in the Intensive Care Unit 24-Item-Revised (FS-ICU-24R) questionnaire (1). Of the original 26 questions, we excluded 15 questions that cannot be answered without the physical presence of the relatives in the ICU. Finally, we used nine questions in the survey (Table S1). Responses were rated on a five-point Likert scale. For validation, we used all available data from the adapted FS-ICU-24R

The adapted version was too short to follow the original manual for the final scoring (2). Therefore, to ensure the accuracy of the adapted FS-ICU-24R, principal component analysis (PCA) and confirmatory factor analysis (CFA) were performed and internal consistency was checked. We have performed a first validation of the adapted version.

**Table S1**. Items of the adapted FS-ICU-24R

| 1. Consideration of your needs? How well the ICU staff showed an interest in your needs. |
| --- |
| 2. Emotional support? How well the ICU staff provided emotional support to you. |
| 3. Frequency of communication with ICU nurses? How often doctors communicated to you about your family member’s condition. |
| 4. Frequency of communication with ICU doctors? How often doctors communicated to you about your family member’s condition. |
| 5. Ease of getting information? Willingness of ICU staff to answer your questions. |
| 6. Understanding of information? How well ICU staff provided you with explanations that you understood. |
| 7. Honesty of information? The honesty of information provided to you about your family member’s condition. |
| 8. Completeness of information? How well ICU staff informed you what was happening to your family member and why things were being done. |
| 9. How included or excluded did you feel in the decision making process? |

Selected Questions from the FS-ICU24R (3)

Descriptive Statistic

The factor analysis and the reliability were calculated with nine items of the adapted FS-ICU-24R. Means and medians are given in Table S2. The answers showed the strongest agreement for item 1 (78%, *N* = 58) and the highest dissatisfaction for item 9 (23%, *N* = 48) (Figure S1).

**Table S2.** Descriptive analysis of the adapted FS-ICU-24R

| Item | 1 | 2 | 3 | 4 | 5 | 6 | 7 | 8 | 9 |
| --- | --- | --- | --- | --- | --- | --- | --- | --- | --- |
| *No.* | 58 | 54 | 56 | 55 | 52 | 57 | 54 | 52 | 48 |
| *Mean*  *SD* | 3.90  1.04 | 3.87  1.05 | 3.63  1.12 | 3.44  1.26 | 3.96  1.07 | 3.74  1.04 | 3.98  .96 | 3.73  1.17 | 3.56  1.43 |
| *Median*  *IQR* | 4.00  1 | 4.00  2 | 4.00  2 | 4.00  1 | 4.00  1 | 4.00  2 | 4.00  2 | 4.00  2 | 4.00  2 |

SD, Standard deviation; IQR, interquartile range

Principal Component Analysis

The structure of the adapted questionnaire was tested using PCA analysis. Both Bartlett’s test (*X^2^* (36) = 320.11, p <0.01) and the Kaiser-Meyer-Olkin Measure of Sampling Adequacy (KMO = 0.93) indicate that the variables are excellently suited for PCA (4). Based on the scree plot (Figure S1) and the considerations of the adapted test version, a one-factor solution was chosen, explaining 76.6% of the variance. The loadings confirm the one-factor solution; all factor loadings are between 0.53 and 0.86 (Table S3).

**Figure S1.** Scree plot

**Table S3.** Item loadings of the adapted FS-ICU-24R

| Item | Loading on factor |
| --- | --- |
| 1. Consideration of your needs? How well the ICU staff showed an interest in your needs. | 0.86 |
| 2. Emotional support? How well the ICU staff provided emotional support to you. | 0.75 |
| 3. Frequency of communication with ICU nurses? How often doctors communicated to you about your family member’s condition. | 0.83 |
| 4. Frequency of communication with ICU doctors? How often doctors communicated to you about your family member’s condition. | 0.81 |
| 5. Ease of getting information? Willingness of ICU staff to answer your questions. | 0.84 |
| 6. Understanding of information? How well ICU staff provided you with explanations that you understood. | 0.73 |
| 7. Honesty of information? The honesty of information provided to you about your family member’s condition. | 0.69 |
| 8. Completeness of information? How well ICU staff informed you what was happening to your family member and why things were being done. | 0.84 |
| 9. How included or excluded did you feel in the decision making process? | 0.53 |

Confirmatory Factor Analysis

The CFA was used to control the model fit indices. Two measures showed a good model fit (Comparative Fit Index [CFI] = 0.99, Tucker-Lewis Index [TLI] = 0.98) and the Root Mean Square Error of Approximation (RMSEA) value is slightly too high (0.067) (5).

Reliability

The calculation of internal consistency revealed a Cronbach’s Alpha of 0.96. Therefore, very good reliability of the adapted FS-ICU-24R can be assumed (6).

The adapted FS-ICU-24R (nine items) appeared to be a validated instrument for measurement of family satisfaction in the given context. Factor analysis was conducted in a specialized ICU population. The adapted FS-ICU24R could be administered to the next of kin of critically ill patients during times of visiting restrictions.

The factor analysis suggested a one-factor solution with good model fits and the findings regarding internal consistency are in line with the assumption. The RMSEA value appeared slightly too high. Altogether, however, we found good fits of the overall model and the adapted FS-ICU-24R seemed to be a valid instrument for assessing family satisfaction in the situation of suspension of ICU visits (7). Nevertheless, further validation including content validity and test-retest reliability analyses should be performed.

# References

1. Wall RJ, Engelberg RA, Downey L, et al. Refinement, scoring, and validation of the Family Satisfaction in the Intensive Care Unit (FS-ICU) survey. Critical care medicine 2007;35(1):271-279.

2. Critical Care Connections Inc., Scoring FS-ICU 24R. [cited 2020 06.12.]Available from: <https://fsicu.org/wp-content/uploads/FS-ICU24R-Scoring-Instructions-.pdf>

3. Wall RJ, Engelberg Ra Fau - Downey L, Downey L Fau - Heyland DK, et al. Refinement, scoring, and validation of the Family Satisfaction in the Intensive Care Unit (FS-ICU) survey. (0090-3493 (Print)).

4. Kaiser HF. A second generation little jiffy. Psychometrika 1970;35(4):401-415.

5. Hu Lt, Bentler PM. Cutoff criteria for fit indexes in covariance structure analysis: Conventional criteria versus new alternatives. Structural Equation Modeling: A Multidisciplinary Journal 1999;6(1):1-55.

6. George D, Mallery P. SPSS for Windows step by step : a simple guide and reference, 17.0 update. 10th ed. ed: Boston : Allyn & Bacon; 2010.

7. Chen F, Curran PJ, Bollen KA, et al. An Empirical Evaluation of the Use of Fixed Cutoff Points in RMSEA Test Statistic in Structural Equation Models. Sociological methods & research 2008;36(4):462-494.
